# Impact of Visual Impairment and Eye diseases on Mortality: the Singapore Malay Eye Study (SiMES)

**DOI:** 10.1038/srep16304

**Published:** 2015-11-09

**Authors:** Rosalynn Grace Siantar, Ching-Yu Cheng, Chui Ming Gemmy Cheung, Ecosse L. Lamoureux, Peng Guan Ong, Khuan Yew Chow, Paul Mitchell, Tin Aung, Tien-Yin Wong, Carol Y. Cheung

**Affiliations:** 1 Singapore Eye Research Institute, Singapore National Eye Centre, Singapore; 2 National Healthcare Group Eye Institute, Tan Tock Seng Hospital, Singapore; 3 Ophthalmology and Visual Sciences Academic Clinical Programme, Duke-NUS Graduate Medical School, National University of Singapore, Singapore; 4 Department of Ophthalmology, Yong Loo Lin School of Medicine, National University of Singapore, Singapore; 5 National Registry of Diseases Office, Ministry of Health, Singapore; 6 Center for Vision Research, University of Sydney, Australia; 7 Department of Ophthalmology and Visual Sciences, The Chinese University of Hong Kong.

## Abstract

We investigated the relationship of visual impairment (VI) and age-related eye diseases with mortality in a prospective, population-based cohort study of 3,280 Malay adults aged 40–80 years between 2004–2006. Participants underwent a full ophthalmic examination and standardized lens and fundus photographic grading. Visual acuity was measured using logMAR chart. VI was defined as presenting (PVA) and best-corrected (BCVA) visual acuity worse than 0.30 logMAR in the better-seeing eye. Participants were linked with mortality records until 2012. During follow-up (median 7.24 years), 398 (12.2%) persons died. In Cox proportional-hazards models adjusting for relevant factors, participants with VI (PVA) had higher all-cause mortality (hazard ratio[HR], 1.57; 95% confidence interval[CI], 1.25–1.96) and cardiovascular (CVD) mortality (HR 1.75; 95% CI, 1.24–2.49) than participants without. Diabetic retinopathy (DR) was associated with increased all-cause (HR 1.70; 95% CI, 1.25–2.36) and CVD mortality (HR 1.57; 95% CI, 1.05–2.43). Retinal vein occlusion (RVO) was associated with increased CVD mortality (HR 3.14; 95% CI, 1.26–7.73). No significant associations were observed between cataract, glaucoma and age-related macular degeneration with mortality. We conclude that persons with VI were more likely to die than persons without. DR and RVO are markers of CVD mortality.

With aging populations, age–related eye diseases and visual impairment (VI) pose substantial public health issues^1^. VI can lead to functional disability^2^,^3^,^4^, loss of independence^5^, reduced social interaction, depression^6^, accidents^7^, falls^8^ and fractures^9^. In addition, as visual acuity is a reflection of one’s functional status and ocular health, the presence of VI may also be a marker of frailty and may hence predict mortality^10^. Several population-based studies have shown a possible link between VI with increased mortality^11^,^12^,^13^,^14^,^15^,^16^,^17^,^18^,^19^. However, there have only been few studies in Asian populations^20^,^21^,^22^, who have different patterns of eye diseases, systemic risk profiles, healthcare knowledge and access to care^23^.

The presence of age-related eye diseases such as cataract, diabetic retinopathy (DR) and age-related macular degeneration (AMD) has also been postulated to be associated with increased mortality risk^15^,^18^,^19^,^22^. The severity of DR, for example, may reflect systemic vascular risk^24^. AMD shares common risk factors with mortality and cardiovascular diseases (CVD)^25^,^26^,^27^,^28^,^29^,^30^,^31^. Similarly, retinal vein occlusion (RVO) has been associated with increased vascular risk and mortality^32^,^33^,^34^. However, the relationship between specific age-related eye diseases with mortality in population-based studies has been mixed. While some studies have reported associations between cataract^15^,^18^,^19^,^20^,^22^,^35^, AMD^15^ and DR^18^,^19^,^22^ with mortality, others have not found independent relationships^12^,^16^,^18^,^19^,^22^,^36^. In addition, only few studies have been conducted in Asia^20^,^21^,^22^ in which AMD and glaucoma, in particular, have different clinical features and possibly underlying pathogenic mechanisms^37^,^38^,^39^,^40^.

Age-related eye diseases and VI is likely to increase substantially in Asia in the next few decades^41^,^42^,^43^. In this study, we describe the relationship of VI and major age-related eye diseases with all-cause and CVD mortality events in a population-based cohort of Asian Malay persons.

## Methods

### Study population

The Singapore Malay Eye Study is a population-based, cohort study, in which 3280 (response rate 78.7%) urban Malay adults aged 40 to 79 years living in Singapore were examined between August 2004 and June 2006. Study design and population details have been described elsewhere^44^. A total of 3273 participants were included in this analysis as visual acuity could not be successfully measured in 7 participants. All study procedures were performed in accordance with the tenets of the Declaration of Helsinki as revised in 1989. Written informed consent was obtained from the subjects and the study was approved by the Singhealth Centralised Institutional Review Board (CIRB approval number R1107/9/2014). Participants underwent a standardized interview, systemic and ocular examination, and laboratory investigations at baseline.

### Assessment of visual impairment

Presenting visual acuity (PVA) (with participants wearing their glasses or contact lenses, if any) and best-corrected visual acuity (BCVA) (with subjective refraction conducted by certified optometrists) were measured using the logMAR number chart (Lighthouse International) at a distance of 4 m. If no numbers were read at 4 m, the participants were moved to 3, 2, or 1 m, consecutively. If no numbers were identified, visual acuity was assessed as counting fingers, hand motions, light perception, or no light perception. BCVA and PVA were used in our analysis, although PVA is likely to be more relevant in the practical setting as this reflects the “true” visual acuity of the study population in their daily activities of living. VI was defined as greater than 0.30 logMAR in the better-seeing eye (US definition)^45^.

### Assessment of age-related eye diseases

Complete ophthalmic examinations of the anterior segment, fundus, and optic discs were conducted at the slit lamp using standardized protocols. Cataracts were assessed from lens photographs using the Wisconsin Cataract Grading System^46^ and defined as nuclear cataract opacity 4 or greater, cortical cataract 25% or greater, posterior subcapsular cataract 5% or greater, or previous cataract surgery. Glaucoma was diagnosed and classified using the International Society Geographical and Epidemiological Ophthalmology scheme based on gonioscopy, optic disc characteristics, and/or visual fields results^47^. The presence of DR was graded from retinal photographs according to a modification of the Airlie House classification system as used in the Early Treatment Diabetic Retinopathy Study^48^. The presence of AMD was graded from retinal photographs according to the Wisconsin Age-Related Maculopathy Grading System^49^. Recent central retinal vein occlusion (CRVO) was characterized by retinal edema, optic disc hyperemia or edema, scattered superficial and deep retinal hemorrhages, and venous dilation. Old CRVO was characterized by occluded and sheathed retinal veins, or vascular anastomosis at the optic disc^50^,^51^. Branch retinal vein occlusion (BRVO) involved a more localized area of the retina in the sector of the obstructed venule and was characterized by scattered superficial and deep retinal hemorrhages, venous dilation, intraretinal microvascular abnormalities, and occluded and sheathed retinal venules^50^,^51^. The presence of any retinal vein occlusion (RVO) was defined as presence of CRVO or BRVO.

### Assessment of other risk factors

Participants underwent a standardized interview for socioeconomic measures (eg, income, education, type of housing), lifestyle risk factors (eg. smoking), medication use, and self-reported history of systemic diseases. Non-fasting venous blood samples were collected for analysis of cholesterol and glucose levels. Hypertension was defined as systolic blood pressure of 140 mm Hg or higher, diastolic blood pressure of 90 mm Hg or higher, or use of antihypertensive medication. Diabetes was defined as a random glucose level of 200 mg/dL (to convert to millimoles per liter, multiply by 0.0555), use of diabetic medication, or a physician’s diagnosis of diabetes. Body Mass Index (BMI) was defined as body mass (kg) divided by the square of the height (m^2^). History of CVD was ascertained through self-reporting (yes or no).

### Assessment of mortality

The unique national registration identity card numbers, together with date of birth and gender, of the 3273 members of the original cohort were matched with mortality records maintained by the National Registry of Diseases Office of Singapore (NRDO). Vital status as of 31 December 2012 was determined for all of the participants. Information on the date of death, all-cause deaths and CVD deaths for the participants were extracted. The underlying cause of death was reported using the International Classification of Diseases 9 codes.

### Statistical Analysis

Time-to-event was calculated for each participant from the date of examination where visual acuity was measured through December 31, 2012. Cox proportional hazard regression was used to investigate the associations of PVA, BCVA, age-related eye diseases and mortality. We constructed 2 multivariable models: 1) adjusted for age and gender; and 2) adjusted for age, gender, socio-economic status, hypertension, smoking, diabetes, BMI and history of CVD. This permits the evaluation of specific ocular diseases and VI on survival while controlling for other risk factors. Finally, each specific ocular condition was added to the model to determine its independent effect on mortality. Hazard ratios and 95% confidence intervals (CI) were presented for stratified PVA and BCVA groups. We regard P values of <0.05 from 2-sided tests to indicate statistical significance. We further tested the interaction between DR and VI with mortality by including cross-product interaction term as independent variable (i.e. DR*VI) in the model. Statistical interactions were deemed significant if the P value for interaction was <0.1, All statistical analyses were performed using STATA version 12.

## Results

### Baseline Characteristics

In total, 3273 participants were included. Of these, 992 (30.3%) met the definition of VI using PVA and 360 (10.9%) met the definition of VI using BCVA.

Table 1 shows the baseline characteristics of participants according to their PVA in the better-seeing eye. Persons with VI tended to be females, have lower socioeconomic status, lower BMI and have a history of CVD, hypertension, diabetes, glaucoma, cataract, retinopathy, AMD, and RVO. Supplementary Table 1 compares the baseline characteristics of participants who met the criteria of VI by PVA but not by BCVA and participants with VI by PVA and BCVA. Persons with VI by both PVA and BCVA tended to be older, have lower socioeconomic status, lower BMI and have a history of CVD, hypertension, diabetes, cataract and late AMD.

### Visual impairment and mortality

**Figure 1:**
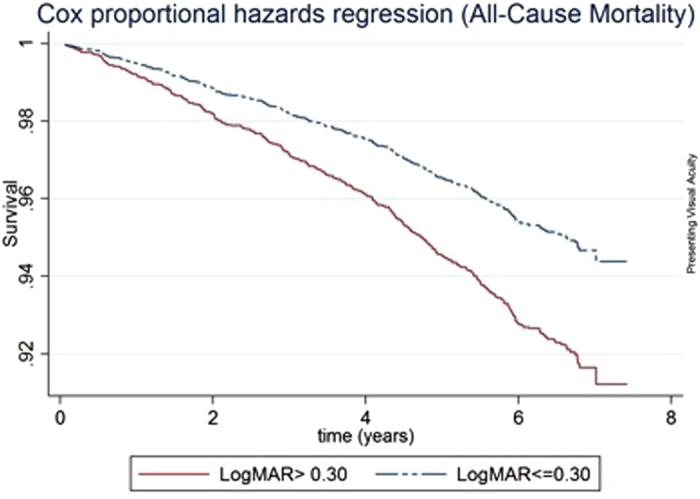
Survival curves for all-cause mortality according to better-eye presenting visual acuity categories, adjusted for age, gender, socio-economic status, diabetes, hypertension, smoking status, BMI and cardiovascular disease.

**Figure 2:**
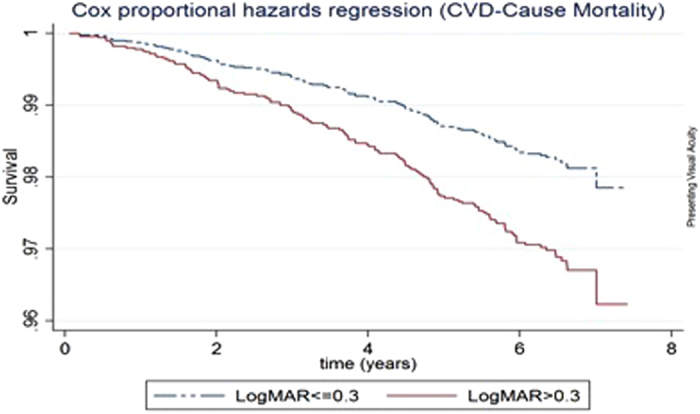
Survival curves for CVD-cause mortality according to better-eye presenting visual acuity categories, adjusted for age, gender, socio-economic status, diabetes, hypertension, smoking status, BMI and cardiovascular disease.

By December 31, 2012 (median follow-up 7.24 years), 398 (12.2%) persons had died in the SiMES cohort. In Cox proportional-hazards models adjusting for age, gender, socioeconomic status, hypertension, diabetes, smoking, BMI and history of CVD, participants with VI (based on PVA) had a higher all-cause mortality rate (hazard ratio [HR], 1.57; 95% confidence interval [CI], 1.25–1.96) and CVD-mortality (HR 1.75; 95% CI, 1.24–2.49) than participants without VI (Table 2). Persons with VI in terms of presenting VA (VA > 0.30) had poorer overall survival (Fig. 1) and higher CVD-cause mortality, compared with those without (Fig. 2). Similarly, persons with VI in terms of BCVA had higher all-cause and CVD-cause mortality. The results were similar when the WHO cut-off for visual impairment (VA < 6/18 in better-seeing eye) was used for presenting and best-corrected VA (Supplementary Table 2).

### Age-related eye diseases and mortality

**Figure 3:**
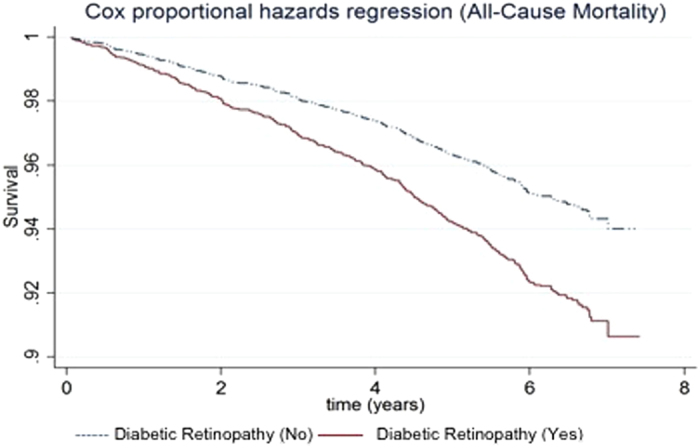
Survival curves for all-cause mortality in subjects with and without diabetic retinopathy, adjusted for age, gender, socio-economic status, hypertension, smoking status, BMI and history of cardiovascular disease.

**Figure 4:**
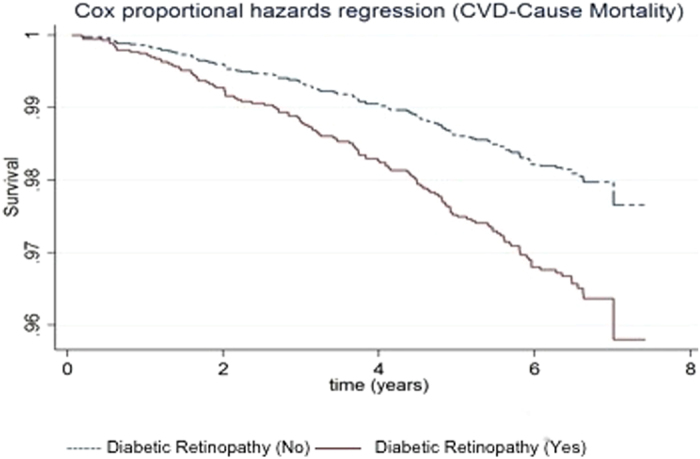
Survival curves for CVD-cause mortality in subjects with and without diabetic retinopathy, adjusted for age, gender, socio-economic status, hypertension, smoking status, BMI and history of cardiovascular disease.

In terms of specific age-related eye disease (Table 3), DR was associated with increased risk of all-cause (HR 1.70; 95% CI, 1.25–2.36) and CVD (HR 1.57; 95% CI, 1.05–2.43) mortality. Participants with DR had poorer survival due to all-cause mortality (Fig. 3) and CVD-cause mortality (Fig. 4) respectively. In addition, RVO was only associated with increased risk of CVD mortality (HR 3.14; 95% CI, 1.26–7.73) but not with all-cause mortality (HR 2.02; 95%CI, 0.91–4.63). None of the other major age-related eye diseases (i.e., cataract, glaucoma and AMD) was statistically significantly associated with mortality. In the stratified analysis (Supplementary Table 3), we found that the association between DR and all-cause mortality was stronger in subjects with VI (p-interaction = 0.035). However, no statistically significant interaction was found with CVD-cause mortality for DR and VI (p-interaction > 0.1).

## Discussion

In this prospective population-based study, persons with VI were more likely to die than persons without VI, even while controlling for other clinical and socio-economical risk factors for mortality. In particular, DR and RVO were associated with increased risk of mortality, particularly CVD mortality.

Our finding that VI is associated with higher risk of mortality is consistent with that found in several studies in the Western population (Table 4)^12^,^15^,^16^,^17^,^18^,^19^ and three in Asian studies^20^,^21^,^22^. As the above population-based studies span across different ethnicities, it is now highly convincing that VI has a close linked relationship with mortality. There are some possible explanations to justify this. First, visual acuity is a reflection of functional status and ocular health. Previous studies have clearly demonstrated that VI leads to functional problems and can contribute to loss of independent mobility^2^,^3^,^4^,^5^, falls^8^, accidents^7^ and psychosocial implications such as depression^6^. These functional problems in turn may contribute to an increased risk of mortality. Second, VI can also be seen as being a marker of aging^52^ and therefore it is not surprising that with aging comes higher incidence of mortality. Having said that, studies have not directly proven a causal relationship between VI and mortality, and thus the exact mechanisms for higher mortality rates associated with VI remain unclear. Our study adds to knowledge of the impact of VI not only on morbidity but also mortality. Reduction of VI such as cataract surgery may also be associated with a reduction in risk of mortality. For example, in the BMES, cataract surgery resulting in the correction of VI resulted in lower long-term mortality risk (HR, 0.55; 95% CI, 0.41–0.73)^53^. Further studies are essentially needed.

In our study, we also observed that major age-related retinal vascular conditions such as DR and RVO were associated with CVD mortality, even after adjusting for potential confounders. DR was also associated with higher risk of all-cause mortality. This positive association of DR with mortality is consistent with other cohort studies^54^,^55^,^56^. The ETDRS study also found that poor visual acuity was associated with mortality in type 1 diabetics, whereas worsening levels of DR was associated with increasing risk of mortality^57^. This is in line with the hypothesis that microvascular diseases such as retinopathy may reflect the macrovascular status in major organs such as the heart and kidney as they may share similar underlying pathophysiology^58^. The presence of DR has also been associated with other risk factors for CVD including hypertension and hyperlipidaemia^59^. When we performed interaction analysis, we found that the association between DR and all-cause mortality was stronger in subjects with VI, indicating that persons presenting with the coexistence of DR and VI were more likely to have increased risk of all-cause mortality. This further highlights the importance of prompt management of DR, which can be sight saving as well as life-saving. Vigilant screening for DR, which can be conveniently achieved in a non-invasive way by direct visualization of the retinal vessels of the eye, and early diagnosis for RVO may therefore play an important role in risk stratification of CVD. In the presence of retinopathy, prompt management of the ocular condition as well as optimization of associated vascular risk factors will be beneficial in disease control and preserving vision, hence reducing VI, which has also been shown to increase risk of mortality as above. We did not find an association between glaucoma, AMD, cataract and increased mortality risk, which was similar to the negative findings in many other studies.

The strengths of this study include its large sample size, long follow-up and standardized diagnostic protocols for cataract, AMD, DR, RVO and glaucoma. To our knowledge, this is also the first study evaluating the association of VI, age-related eye disease and mortality in an Asian Malay population. Limitations of our study include the possibility of some of the results being due to chance as there are a large number of confounding factors involved and not all may be accounted for. Further studies are also needed to investigate if VI has a direct or indirect causal effect on mortality. Longer duration of follow up may also be useful in confirming these associations. We also did not have data on the time of onset of cataract and time of surgery, which may confound the relationship between the presence of cataract and mortality, and whether cataract surgery had an effect on mortality risk.

In conclusion, persons with VI were more likely to die than persons without VI. The presence of DR and RVO were, in particular, markers of increased mortality and CVD risk.

## Additional Information

**How to cite this article**: Siantar, R. G. *et al.* Impact of Visual Impairment and Eye diseases on Mortality: the Singapore Malay Eye Study (SiMES). *Sci. Rep.* **5**, 16304; doi: 10.1038/srep16304 (2015).

## Supplementary Material

Supplementary Information

## Figures and Tables

**Table 1:** Baseline Characteristics of Study Participants by Presenting Visual Acuity Categories in the Better Eye in The Singapore Malay Eye Study.

Characteristics	Presenting VA, LogMAR scores		P value†
	VA <= 0.30(n = 2281)	VA > 0.30(n = 992)	
Age (yrs)	55.82 (10.23)	64.95 (10.23)	<0.001
Gender, Male	1173 (51.42)	400 (40.32)	<0.001
Low socioeconomic status, yes	395 (17.45)	391 (40.06)	<0.001
Smoking			<0.001
- Current	512 (22.48)	150 (15.21)	
- Past	406 (17.82)	188 (19.07)	
- Never	1360 (59.7)	648 (65.72)	
BMI	26.59 (5.08)	25.80 (5.15)	<0.001
Hypertension, yes	1444 (63.31)	797 (80.34)	<0.001
Diabetes, yes	475 (21.50)	291 (30.25)	<0.001
History of cardiovascular disease	219 (9.63)	147 (14.92)	<0.001
- Previous Stroke, yes	36 (1.58)	45 (4.55)	<0.001
- Previous AMI, yes	111 (4.87)	101 (10.22)	<0.001
Glaucoma, yes	86 (3.77)	64 (6.45)	0.001
Any cataract, yes	695 (34.04)	639 (72.53)	<0.001
Nuclear cataract, yes	238 (11.69)	424 (49.53)	<0.001
Cortical cataract, yes	516 (24.58)	412 (48.36)	<0.001
PSC, yes	198 (19.51)	272 (31.81)	<0.001
Any retinopathy, yes	275 (12.09)	145 (14.75)	0.037
Diabetic retinopathy, yes	172 (36.46)	106 (36.93)	0.873
Retinopathy (non-diabetic), yes	103 (5.95)	39 (5.86)	0.928
Any AMD, yes	105 (4.62)	78 (7.91)	<0.001
- early AMD, yes	96 (4.22)	64 (6.49)	0.006
- late AMD, yes	9 (0.40)	14 (1.42)	0.001
Retinal vein occlusions, yes	11 (0.48)	12 (1.22)	0.022

AMD = Age-related Macular Degeneration; AMI = Acute Myocardial Infection; BMI = Body Mass Index; PSC = Posterior Subcapsular Cataracts; VA = Visual Acuity; LogMAR = Logarithm of the Minimum Angle of Resolution Scores. Data are either presented in mean (standard deviation) or number (%) for continuous and categorical variables respectively. ^†^p-value for t test or chi-square test where appropriate.

**Table 2:** Proportional Hazard Models of All-Cause Mortality and CVD-Cause Mortality by Better-Eye Presenting Visual Acuity and Best-Corrected Visual Acuity in All Participants.

	All-Cause Mortality Hazard Ratio (95% CI)				CVD-Cause Mortality Hazard Ratio (95% CI)			
	No.	No. (%)	Model 1*	Model 2**	No.	No. (%)	Model 1*	Model 2**
Presenting Visual Acuity, LogMAR (Snellen Equivalent)								
All participants	3273	398 (12.2)			3273	167 (5.10)		
VA <= 0.30(Snellen ≥20/40)	2281	174 (7.63)	Referent	Referent	2281	67 (2.94)	Referent	Referent
VA > 0.30(Snellen < 20/40)	992	224 (22.6)	1.65 (1.33, 2.04)‡	1.57 (1.25, 1.96)‡	992	100 (10.08)	1.89 (1.35, 2.64)†	1.75 (1.24, 2.49)†
Best-Corrected Visual Acuity, LogMAR (Snellen Equivalent)								
All participants	3273	398 (12.2)			3273	167 (5.10)		
VA <= 0.30(Snellen ≥20/40)	2913	277 (9.51)	Referent	Referent	2913	115 (3.95)	Referent	Referent
VA > 0.30(Snellen < 20/40)	360	121 (33.6)	1.67 (1.32, 2.11)‡	1.46 (1.14, 1.88)‡	360	52 (14.44)	1.62 (1.13, 2.33)‡	1.49 (1.02, 1.88)†

CI = Confidence Interval; VA = Visual Acuity; LogMAR = Logarithm of the Minimum Angle of Resolution. Model 1: Adjusted for age and gender. Model 2: Adjusted for age, gender, socio-economic status, diabetes, hypertension, smoking status, BMI and cardiovascular disease. ^†^P < 0.05; ^‡^P < 0.001.

**Table 3:** Proportional Hazard Models of All-Cause Mortality and CVD-Cause Mortality by Age-related Eye Diseases in All Participants.

Ocular Diseases	All-Cause Mortality Hazard Ratio (95% Cl)				CVD-Cause Mortality Hazard Ratio (95% CI)			
	No.	No. (%)	Model 1	Model 2	No.	No. (%)	Model 1	Model 2
Glaucoma, yes	150	34 (22.7)	1.23 (0.87,1.76)	1.27 (0.87,1.85)	150	12 (8.00)	0.97 (0.54,1.75)	0.98 (0.55,1.82)
Cataract, yes	1334	248 (18.6)	1.23 (0.88, 1.73)	1.13 (0.82,1.60)	1334	96 (7.20)	0.95 (0.57,1.58)	0.91 (0.51,1.43)
- Nuclear cataract, yes	662	160 (24.2)	1.09 (0.82, 1.44)	1.22 (0.91,1.60)	662	62 (9.37)	0.94 (0.61,1.46)	1.02 (0.65,1.63)
- Cortical cataract, yes	928	172 (18.5)	1.13 (0.90,1.43)	1.10 (0.88,1.39)	928	66 (7.11)	1.03 (0.71,1.50)	1.01 (0.70,1.48)
- PSC cataract, yes	470	102 (21.7)	1.06 (0.83,1.36)	1.08 (0.83,1.40)	470	37 (7.87)	0.92 (0.61,1.37)	0.98 (0.66,1.44)
Any retinopathy, yes	420	87 (20.7)	2.17 (1.17, 2.76)‡	1.55 (1.19, 2.02)‡	420	43 (10.24)	2.53 (1.78, 3.59)‡	1.16 (1.13, 2.44)†
Diabetic retinopathy, yes	269	73 (27.1)	1.85 (1.35,2.52)‡	1.70 (1.25,2.36)‡	269	35 (13.01)	1.70 (1.09,2.67)†	1.57 (1.05,2.43)†
Retinopathy, yes (non-diabetic)	142	13 (9.2)	1.12 (0.64,1.96)	1.06 (0.63,1.88)	142	8 (5.63)	1.93 (0.93,4.00)	1.73 (0.81,3.67)
AMD, yes	183	40 (21.9)	1.02 (0.73,1.42)	1.05 (0.74,1.46)	183	17 (9.29)	1.03 (0.62,1.72)	0.98 (0.57,1.72)
- early AMD, yes	160	33 (20.6)	1.03 (0.72,1.48)	1.08 (0.71,1.55)	160	126 (10.0)	1.22 (0.72,2.05)	1.34 (0.81,2.30)
- late AMD, yes	23	7 (30.4)	0.97 (0.46,2.05)	0.82 (0.34,1.80)	23	1 (4.35)	0.32 (0.04,2.27)	NA
Retinal vein occlusions, yes	23	7 (30.4)	2.33 (1.10,4.92)†	2.02 (0.91,4.63)	23	6 (26.09)	4.72 (2.08,10.7)‡	3.14 (1.26,7.73)†

Model 1: Adjusted for age and gender. Model 2: Adjusted for age, gender, socio-economic status, hypertension, smoking, BMI and cardiovascular disease. ^†^P < 0.05; ^‡^P < 0.001.

**Table 4:** Summary table of main findings from population-based studies on the relationship between visual impairment, age-related eye diseases and all-cause and CVD mortality.

Study	VisualImpairment	Cataract	DR	Glaucoma	AMD	Retinal Vein Occlusion
White populations						
Beaver Dam Eye Study, USA^23^,^39^	+	+	+	−	−	−
	1.24 (1.04,1.48)	1.16 (1.03, 1.32)	1.36 (1.14, 1.63)	1.04 (0.84, 1.28)	0.97 (0.87, 1.07)	1.1 (0.6, 2.0) (CVD related mortality)
Blue Mountains Eye Study, Australia^18^,^24^,^39^	Borderline	+	Borderline	−	−	−
	1.27 (0.98, 1.66)	1.32 (1.11, 1.57)	1.6 (1.0, 2.7)	Data not shown	1.03 (0.84.1.26)	1.2 (0.7, 2.1)
	+	+			−	(CVD related mortality)
	2.92 (1.61, 5.33) (age < 75)	1.67 (1.24, 2.25) (CVD related mortality)			0.93 (0.68, 1.28) (CVD related mortality)	
	−					
	1.1 (0.74, 1.64) (CVD related mortality)					
Visual Impairment Project, Melbourne, Australia^17^	+	−	NR	−	−	NR
	2.34 (1.03, 5.32)	0.81 (0.53, 1.26)		1.15 (0.63, 2.11)	1.36 (0.91, 1.94)	
Age-Related Eye Disease Study, USA^20^	+	+	NR	NR	+	NR
	1.36 (1.12, 1.65)	1.40 (1.12, 1.75)			1.44 (1.08, 1.86)	
		−			+	
		1.19 (0.82, 1.73) (CVD related mortality for nuclear cataract)			1.92 (1.18, 3.12) (CVD related mortality)	
Medical Research Council Study (MRCS)^21^	+	−	NR	NR	−	NR
	1.17 (1.07, 1.27)	1.04 (0.84, 1.28)			1.01(0.81, 1.25)	
		−			−	
		0.98 (0.65, 1.48) (CVD related mortality)			1.03 (0.72, 1.45) (CVD related mortality)	
Rotterdam Eye Study^43^	NR	−	NR	−	−	NR
		0.94 (0.74, 1.21)		0.39 (0.10, 1.55)	0.94 (0.52, 1.68)	
Salisbury Eye Evaluation Project^22^,^42^	+	+	NR	NR	NR	NR
	1.05 (1.01, 1.09)	2.23 (1.26, 3.95) (mixed nuclear)				
Asian populations						
Singapore Malay Eye Study, Singapore	+	−	+	−	−	Borderline
	1.66 (1.33, 2.07)	Any cataract 1.16 (0.84, 1.62)	1.74 (1.27, 2.38)	1.26 (0.87, 1.83)	1.02 (0.72, 1.45)	2.03 (0.91, 4.59)
		Nuclear 1.19 (0.89, 1.59)				+
		Cortical 1.08 (0.85, 1.37)				3.05 (1.22, 7.60) (CVD related mortality)
		PSC 1.04 (0.81, 1.35)				
Beijing Eye Study, China^27^	−	+/−	+	-	-	-
	Data not shown	Nuclear 1.29 (1.10, 1.52)	2.26	Data not shown	Data not shown	Data not shown
		Data not shown for cortical and subcapsular	(1.34, 3.81)			
Tanjong Pagar Study, Singapore^26^	+	−	NR	-	NR	NR
	2.9 (1.4, 6.3)	Data not shown		Data not shown		
Andhra Pradesh Eye Disease Study, India^25^	+	+	NR	NR	NR	NR
	1.4 (1.2, 1.7)	1.58 (1.3, 1.9)				

Data shown are either hazard ratios or odds ratios from multivariate analysis. +: positive association, −: negative association, NR: not reported.
