# Supplementary material for: Impact of Visual Impairment and Eye diseases on Mortality: the Singapore Malay Eye Study (SiMES)
Source: Sci Rep. 2015 Nov 9;5:16304. doi: 10.1038/srep16304 (PMC4637872; doi:10.1038/srep16304)
Supplement: Supplementary Information [file srep16304-s1.doc]

**Impact of Visual Impairment and Eye diseases on Mortality: the Singapore Malay Eye Study (SiMES)**

Rosalynn Grace Siantar (1, 2), Ching-Yu Cheng (1,3,4), Chui Ming Gemmy Cheung (1,3), Ecosse L Lamoureux (1,3), Peng Guan Ong (1), Khuan Yew Chow(5), Paul Mitchell (6), Tin Aung (1,3,4), Tien Yin Wong (1,3,4), Carol Y Cheung* (1,3,4,7)

1. Singapore Eye Research Institute, Singapore National Eye Centre, Singapore

2. National Healthcare Group Eye Institute, Tan Tock Seng Hospital, Singapore

3. Ophthalmology and Visual Sciences Academic Clinical Programme, Duke-NUS Graduate Medical School, National University of Singapore, Singapore

4. Department of Ophthalmology, Yong Loo Lin School of Medicine, National University of Singapore, Singapore

5. National Registry of Diseases Office, Ministry of Health, Singapore

6. Center for Vision Research, University of Sydney, Australia

7. Department of Ophthalmology and Visual Sciences, The Chinese University of Hong Kong

Correspondence to: Dr. Carol Y. Cheung (Email: cheungcarol@gmail.com)

Address: Singapore Eye Research Institute, 20 College Road, The Academia, Discovery Tower, Level 6, Singapore 169856, Phone: (65) 6575 7237, Fax: (65) 6323 1903

| **Supplementary Table 1.** Baseline characteristics of participants with VI by PVA but not BCVA and participants with VI by PVA and BCVA. | | | | | |
| --- | --- | --- | --- | --- | --- |
| **Characteristics** | **PVA > 0.3 & BVA <=0.3** | | **PVA & BVA > 0.30** | | **P value†** |
|  | (n=632) | | (n=360) | |  |
| Mean age (yrs) | 61.89 (10.18) | | 70.48 (7.68) | | <0.001 |
| Gender, Male | 268 (42.01) | | 132 (37.29) | | 0.147 |
| Low socioeconomic status | 210 (33.49) | | 181 (51.86) | | <0.001 |
| Smoking |  | |  | |  |
| -Current | 406 (63.94) | | 242 (68.95) | | 0.001 |
| -Past | 116 (18.27) | | 34 (9.69) | |  |
| -Never | 113 (17.8) | | 75 (21.37) | |  |
| BMI | 25.94 (4.98) | | 25.57 (5.46) | | <0.001 |
| Diabetes, yes | 160 (26.02) | | 131 (37.75) | | <0.001 |
| History of cardiovascular disease | 83 (13.09) | | 64 (18.23) | | 0.03 |
| - Previous Stroke, yes | 21 (3.3) | | 24 (6.82) | | 0.011 |
| Previous AMI, yes | 60 (9.43) | | 41 (11.65) | | 0.271 |
| Glaucoma, yes | 41 (6.43) | | 23 (6.51) | | 0.965 |
| Existing cataract, yes | 333 (59.12) | | 306 (96.23) | | <0.001 |
| Nuclear cataract, yes | 175 (31.7) | | 249 (81.91) | | <0.001 |
| Cortical cataract, yes | 220 (39.5) | | 192 (65.08) | | <0.001 |
| PSC, yes | 114 (20.61) | | 158 (52.32) | | <0.001 |
| Retinopathy, yes | 96 (15.09) | | 49 (14.12) | | 0.681 |
| Diabetic retinopathy, yes | 65 (40.88) | | 41 (32.03) | | 0.123 |
| Retinopathy (non-diabetic), yes | 31 (6.83) | | 8 (3.77) | | 0.118 |
| Any AMD, yes | 44 (6.91) | | 34 (9.74) | | 0.115 |
| -early AMD, yes | 40 (6.23) | | 24 (6.88) | | 0.716 |
| - late AMD, yes | 4 (0.63) | | 10 (2.87) | | 0.005 |
| Retinal vein occlusions, yes | 7 (1.1) | | 5 (1.43) | | 0.648 |
| AMD = Age Macular Degeneration; AMI = Acute Myocardial Infection; PSC =  Posterior Subcapsular Cataracts | | | | | |
| VA= Visual Acuity; LogMAR= Logarithm of the Minimum Angle of Resolution Scores; BMI = Body Mass Index | | | | | |
| Data are either presented in mean (s.d.) or number(%) for continuous and categorical variables respectively. | | | | | |
| † p value for t test or chisquare test where appropriate. | |  | |  | |

**Supplementary Table 2.** Proportional Hazard Models of All-Cause Mortality and CVD-Cause Mortality by Better-Eye Presenting Visual Acuity and Best-Corrected Visual Acuity by WHO criteria in All Participants

|  | **All-Cause Mortality Hazard Ratio (95% CI)** | | | |  | **CVD-Cause Mortality Hazard Ratio (95% CI)** | | | |
| --- | --- | --- | --- | --- | --- | --- | --- | --- | --- |
|  | No. | No. (%) | Model 1* | Model 2** |  | No. | No. (%) | Model 1* | Model 2** |
| **Presenting Visual Acuity, LogMAR (Snellen Equivalent)** | | | | | | | | | |
| All participants | 3273 | 398 (12.2) |  |  |  | 3273 | 167  (5.1) |  |  |
| VA<= 0.48  (Snellen ≥6/18) | 2803 | 275 (9.81) | Referent | Referent |  | 2803 | 112  (4.00) | Referent | Referent |
| VA>0.48  (Snellen <6/18) | 470 | 123 (26.2) | 1.48  (1.18, 1.86)‡ | 1.72  (1.28, 2.32)‡ |  | 470 | 55 (11.7) | 1.59  (1.13, 2.25)† | 1.51  (1.08, 2.41)† |
| **Best-Corrected Visual Acuity, LogMAR (Snellen Equivalent)** | | | | | | | | | |
| All participants | 3273 | 398 (12.2) |  |  |  | 3273 | 167  (5.1) |  |  |
| VA<= 0.48  (Snellen ≥6/18) | 2803 | 275 (9.81) | Referent | Referent |  | 3133 | 144  (4.60) | Referent | Referent |
| VA> 0.48  (Snellen <6/18) | 470 | 123 (26.2) | 1.48  (1.17, 1.87)‡ | 1.63  (1.19, 2.22)‡ |  | 140 | 23  (16.4) | 1.55  (1.08, 2.22)† | 1.35  (0.83, 2.19) |
| CI= Confidence Interval; VA = Visual Acuity; LogMAR = Logarithm of the Minimum Angle of Resolution.  Model 1: Adjusted for age and gender  Model 2: Adjusted for age, gender, socio-economic status, diabetes, hypertension, smoking status, BMI and cardiovascular disease.  †P<0.05; ‡P<0.001 | | | | | | | | | |

**Supplementary Table 3.** Interaction analysis of Diabetic Retinopathy and Visual Impairment with All-cause and CVD-cause Mortality

|  | **All-cause mortality** | | | |
| --- | --- | --- | --- | --- |
|  | No. at risk | N (%) | Multivariable HR (95%CI) |  |
| **Combined Categories** |  |  |  |  |
| DR (No) and BCVA<=0.30 | 402 | 61 (15.2%) | Reference |  |
| DR (No) and BCVA>0.30 | 89 | 33 (37.1%) | 1.49 (0.94, 2.37) |  |
| DR (Yes) and BCVA<=0.30 | 228 | 53 (23.3%) | 1.58 (1.08, 2.31)† |  |
| DR (Yes) and BCVA>0.30 | 41 | 20 (48.8%) | 2.79 (1.55, 5.01)‡ |  |
|  |  |  |  |  |
| DR (No) and PVA<=0.30 | 310 | 39 (12.6%) | Reference |  |
| DR (No) and PVA>0.30 | 181 | 55 (30.4%) | 1.57 (1.01, 2.45)† |  |
| DR (Yes) and PVA<=0.30 | 163 | 35 (21.5%) | 1.71 (1.07, 2.73)† |  |
| DR (Yes) and PVA>0.30 | 106 | 38 (35.9%) | 2.35 (1.44, 3.84)‡ |  |

|  | **CVD-cause mortality** | | | |
| --- | --- | --- | --- | --- |
|  | No. at risk | N (%) | Multivariable HR (95%CI) |  |
| **Combined Categories** |  |  |  |  |
| DR (No) and BCVA<=0.30 | 402 | 29 (7.2%) | Reference |  |
| DR (No) and BCVA>0.30 | 89 | 15 (16.9%) | 1.36 (0.69, 2.71) |  |
| DR (Yes) and BCVA<=0.30 | 228 | 27 (11.8%) | 1.60 (0.93, 2.75) |  |
| DR (Yes) and BCVA>0.30 | 41 | 8 19.5%) | 1.83 (0.73, 4.56) |  |
|  |  |  |  |  |
| DR (No) and PVA<=0.30 | 310 | 20 (6.5%) | Reference |  |
| DR (No) and PVA>0.30 | 181 | 24 (13.3%) | 1.37 (0.73, 2.64) |  |
| DR (Yes) and PVA<=0.30 | 163 | 16 (9.8%) | 1.37 (0.69, 2.72) |  |
| DR (Yes) and PVA>0.30 | 106 | 19 (17.9%) | 2.31 (1.16, 4.60)† |  |

**P-interaction (DR *VI) = 0.035 for all-cause mortality and > 0.1 for CVD-cause mortality**

**Supplementary Table 4.** Proportional Hazard Models of All-Cause Mortality and CVD-Cause Mortality by Age-related Eye Diseases in All Participants Stratified by Gender

| **Ocular Diseases** |  | **Hazard Ratio (95% CI)** | | | |
| --- | --- | --- | --- | --- | --- |
|  |  | Male (all-cause mortality) | Female (all-cause mortality) | Male (CVD-cause mortality) | Female (CVD-cause mortality) |
| Glaucoma, yes |  | 1.29 (0.80, 2.07) | 1.31 (0.72, 2.40) | 0.86 (0.37, 1.99) | 1.12 (0.45, 2.83) |
| Cataract, yes |  | 0.97 (0.64, 1.46) | 1.53 (0.88, 2.68) | 0.65 (0.36, 1.17) | 1.59  (0.63, 4.00) |
| - Nuclear cataract, yes |  | 1.15 (0.79, 1.70) | 1.20 (0.77, 1.88) | 0.90 (0.50, 1.62) | 1.32  (0.65, 2.68) |
| - Cortical cataract, yes |  | 1.00 (0.73, 1.37) | 1.18 (0.81, 1.70) | 0.87 (0.54, 1.43) | 1.22  (0.68, 2.19) |
| - PSC cataract, yes |  | 1.03 (0.73, 1.45) | 1.01 (0.69, 1.50) | 0.74 (0.42, 1.31) | 1.29  (0.71, 2.34) |
| Any Retinopathy, yes |  | 1.53 (1.08, 2.19)† | 1.54 (1.04, 2.28)† | 1.50 (0.89, 2.52) | 1.84  (1.03, 3.30)† |
| Diabetic retinopathy, yes |  | 1.92 (1.23, 2.99)‡ | 1.60 (1.02, 2.50)† | 1.36 (0.72, 2.60) | 1.80  (0.95, 2.41) |
| Retinopathy, yes (non-diabetic) |  | 1.13 (0.75, 1.71) | 1.39 (0.56, 3.46) | 1.75 (0.75, 4.14) | 2.21  (0.50, 9.65) |
| AMD, yes |  | 1.15 (0.73, 1.82) | 0.74 (0.36, 1.51) | 1.03 (0.54, 1.97) | 0.81  (0.29, 2.29) |
| -early AMD, yes |  | 1.05 (0.16, 2.37) | 0.90 (0.44, 1.83) | 1.46 (0.77, 2.77) | 1.06  (0.38, 2.93) |
| - late AMD, yes |  | NA | NA | NA | NA |
| Retinal vein occlusions, yes |  | 1.72 (0.43, 7.01) | 1.92 (0.69, 5.32) | 5.20  (1.26, 21.4)‡ | 2.18  (0.65, 7.35)† |
